# The Foreign Journals: Surgery

**Published:** 1840-01

**Authors:** 


### SURGERY.

*On the Treatment of Varix of the Inferior Extremities. 1. By Pins. 2. By Caustic Potass. 3. By the Combination of these Means.* By M. Bonnet, First Surgeon of the Hôtel Dieu de Lyon.

[We doubt not that at the present time, when much is said about various methods of treating varicose veins, an attempt such as the following, to ascertain the value of a particular treatment, not only from its immediate but from its subsequent effects, will be very acceptable to many of our readers. In this, as in some other operations of surgery, a partiality may be acquired for a particular method of operating, from something which is attractive in the operation itself and its immediate consequences, too little regard being paid to its ultimate effect upon the disease, the complete remedying of which should be the paramount object of all operations.]

In 1834, I commenced, says M. Bonnet, to study the subject of the radical cure of varices. In the following year, I employed pins in sixteen cases, in which the veins were in relief beneath the skin, and caustic potass in two cases of females, in which the dilated veins were lost in a large quantity of fat. The last two were cured, as well as all those who were treated with pins, and who were in such a condition as to render a radical cure certain. Both methods appeared to me equally useful. But as hemorrhage had occurred through the eschars at the time that the caustic had opened the veins, and as no serious accident had followed the use of pins, I came to the conclusion that the operation by pins was to be preferred. But I determined to watch the progress of these cases; and the consequence was, that I was disappointed in the effect of the pins. All the patients whom I saw again, after having remained well during a time varying from one to six months, were reaffected with varix, with as much intensity as before the operation, and this not only in the secondary divisions of the veins, but in the trunk of the saphena, on which the larger number of pins had been applied, and where it appeared that the obliteration would be permanent. In the two cases treated by caustic potass, there was no return of the disease: one of these was seen fourteen months after the treatment, the other several years afterwards. From these facts I was led to infer, that the pins produced but a temporary obliteration; but that caustic potass determined a permanent closure, its use, however, being attended with risk of hemorrhage. I considered, therefore, that if I placed pins at intervals upon the saphena, and cauterized the vein between them, I should obtain a temporary obliteration by the pins, such as would prevent hemorrhage, and a permanent obliteration by caustic, such as would effect a cure. I employed this treatment on nine patients, in 1837, but these cases I never saw again. But, in 1838, I treated a man in whom the ulcerated varicose veins gave rise to abundant hemorrhage. I had lost confidence in pins, and found the combination of pins and caustic too complex. I had, therefore, recourse to caustic alone, believing that bleeding might be controlled by compression and position. The result of the practice, in this case, having justified my expectations, I resumed a practice, which I immediately employed on a new series of twelve cases, the course of which more and more confirmed me in the idea, that the treatment of varices by caustic alone is, of all the methods which I employed, the most simple in its application, the least uncertain in its effects, and that which secures the most complete and the most permanent cure.

Before further considering the question of treatment, which is the main object of this paper, I would notice a fact in morbid anatomy relative to varices, and a symptom from which their importance and the effects of treatment upon them may be inferred. I speak of those tumours which stand in the same relation to veins that spontaneous aneurisms do to arteries, and of the undulation which may be communicated to the blood in varicose veins, in a contrary direction to that which happens when the valves are entire, and the blood takes its normal course.

*Varicose Tumours analogous to Spontaneous Aneurisms.*—I removed one of these tumours from the course of the internal saphena vein: it contained a quantity of liquid blood, with soft and blackish clots; its fibrous walls were perfectly smooth on their internal surface, and its cavity communicated with that of the vein by an opening, three or four lines in diameter, which surrounded the small portion of the vein which I had detached. This tumour was like an aneurism; its walls were continuous with those of the vessel on the side of which it was situated, and the cavities of the one and of the other communicated by a narrow aperture. I have since met with this affection in two instances, and each time on the crural portion of the internal saphena vein. On percussing one of these tumours, the blood was made to flow backwards in the dilated vessel, and on placing the hand upon the other, a distinct pulsation was felt, which pulsation was also visible. This reflux, this pulsation, seems to me to be the distinctive character of the tumours; because it shows that the fluid contained in them communicates freely with that in the veins. This form of varix has been very little mentioned by authors. The two cases just mentioned were treated simply by passing a pin through the bloody mass: it was secured by the twisted suture, and not removed, in either case, for eight days. The blood began to coagulate on the second day; on the eighth, the swelling had become hard, and in about a month it was very hard, and of the size of a small nut. This I regard as a successful issue of the treatment of these tumours by means of pins. The undulation of the blood caused by percussion of varicose veins should be very carefully attended to, both as a mean of judging of the difficulty which may attend the radical cure of varix, and especially to ascertain whether or no, when pins have been employed, they have been employed properly; for if the pins properly compress the opposite sides of a vein, the undulation of blood stops at the place so compressed. This sign should also be attended to at the conclusion of the treatment, as a mean of judging if the treatment requires to be renewed.

*Treatment of Varices by Pins alone.*—Velpeau, Davat, Jobert, who have written on the treatment of varices by pins, have not specified the cases in which they necessarily fail, and in such cases as failure does not happen, to what extent the cure is permanent; nor have they insisted on the principles on which this treatment is founded. These defects it is my intention to supply.

*In the treatment of Varices, it is necessary to obliterate the veins in several points, separated from each other by short intervals.*—The necessity of this is admitted, depending, as it does, on the numerous anastomoses of the venous trunks. It is easy to find, in various works, instances of continuance or recurrence of varix, after the obliteration of a vein in one place alone, whether this obliteration was spontaneous, or artificially effected. On the other hand, in addition to written evidence of an opposite character, all the observations which I have made on the obliteration of veins, either by pins or by caustic potass, have but confirmed me in the opinion, that veins should be obliterated in several points. I have always observed, that the coagulation of the blood, and the contraction of the vein, took place only in the vicinity of the obliterated points; that the divisions of the veins distant from those which the operation had rendered almost impermeable to blood, remained almost as dilated as before the operation. I consequently have been in the habit of obliterating at as many points as possible, placing from four to ten pins on the same individual, some on the course of the internal saphena, from three to four inches apart, others at the point of junction, or upon the course of the principal divisions connected with it.

*Methods of placing the Pins.*—There are three principal methods. That of M. Davat consists in plunging the pin upon the middle part of the vein which is to be obliterated, passing it through from the superficial to the deep part, and again in a contrary direction. When the point of the needle protrudes, it is fixed by means of a thread, twisted as in the twisted suture, and it is allowed to remain until the parietes of the vein, in contact with one another at the divided part, become inflamed and adherent. This plan is easily enough effected by means of common pins, when the part to be transfixed takes an oblique direction in relation to the axis of the limb. In this case, after having transfixed the vein, the head of the pin being depressed, its point is passed for two or three times behind the vessel, which is again transfixed with ease, from within outwards. But when the vein runs parallel to the limb, as is commonly the case in the thigh, it is difficult, without giving an oblique direction to the pin, to pass it properly, so that after having transfixed it inwards, the pin may not enter it in coming outwards, but may pass behind it. The method of M. Velpeau consists in passing a pin transversely beneath a vein, without wounding its parietes, securing it afterwards by means of the twisted suture. M. Fricke simply transfixes the vein with a pin, and allows it to act as a seton in the production of adhesive inflammation. The method of M. Davat appears the most efficient of these three: it ensures inflammation, compression, and the contact of the parietes of the vein, in those parts which the pins have divided. M. Davat considers that it is very important to slightly divide the membranes of the vein, before putting them into contact. He thinks M. Velpeau's method insufficient, that it simply produces coagulation which may not be durable. He dissected the jugular vein of three dogs, which he had operated on in the manner described by M. Velpeau. In one case, the pins were withdrawn on the eleventh day; in the other two cases, on the ninth. In the first case, the vein was thickened for the extent of an inch, a fibrous cylinder occupying its cavity, which was contracted but not obliterated. In the other two cases, the course of the blood was re-established, the veins being simply thickened. Other experiments of M. Davat show that obliteration depends upon adhesive inflammation, that the cicatrices are durable, and not dissipated with the other effects of the inflammation.

*Period during which I have left the pins in place.*—I have never waited for the establishment of suppuration before removing the pins. When the red tumefaction and pain around the pin commenced, I cut the thread, and as soon as the swelling and redness were very marked, and the pain such as to disturb sleep, I removed the pin. The time required for the production of these symptoms varied from three to fifteen days. In one case only, having neglected to take away a pin which caused great pain, and which had produced, on the fourth day, a swelling, the surface of which was somewhat larger than a sixpence, an active inflammation occurred in the upper half of the leg. This was of a phlegmonous character, requiring very active means to prevent suppuration. This, and other cases, induced me to remove the pins before suppurative inflammation took place.

*Results obtained by treatment of Varices by Pins alone.*—These cases may be thus classed: 1. Varices without ulceration of the vein, without œdema, the vessels being easily visible, and capable also of being felt throughout their whole course. 2. Varices very much folded on themselves, resembling, in this respect, the intestines. 3. Varices accompanied by œdema, and losing themselves in a quantity of fat. Eleven patients belonged to the first class; two of these were more than sixty-three years of age, and were debilitated. The cure was not even momentary, the blood did not coagulate in the interspaces of the pins, and before they left the hospital, the obliteration, the existence of which was known by the stoppage of the undulation, even after the removal of the pins, was completely destroyed. These facts are not astonishing. In advanced life, the blood is indisposed to coagulate, adhesions are indisposed to form; and no attempt should be made to cure varices after the age of sixty years. The other nine were, with one exception, under fifty-four years of age: they left the hospital quite cured; the blood was coagulated in the whole course of the varicose vessels; these were diminished in size, and a very long walk did not cause swelling of the veins. But, of these nine, I saw five again. In two of these, the veins began to enlarge and to become permeable, as soon as the patients resumed their occupations; in two others, two or three months afterwards, and in the fifth, six months after his having left the hospital. One or two months more were required in each case, before the dilatation was as much as before the operation. The patients of the second class were almost completely cured: some few venous branches only swelled whilst walking. In one of these, fifteen months afterwards, the internal saphena and its branches had become as large as before the use of the pins. In the two other cases, in whom the veins were very large, lost in a great quantity of fat, and spread in large number over the whole leg and instep, although in one of these I applied twelve pins, and in the other fourteen, which were not withdrawn until from the eleventh to the fifteenth day, only a momentary benefit was obtained. One of these was sixty, and the other forty-eight years of age. The same failure occurred in two patients, whose varices were imbedded in fat, and were attended with œdema. Of the twelve cases regarded as cured on leaving the hospital, the six whom I saw afterwards suffered a relapse, after a longer or shorter period. There were among these some whose veins were most acutely inflamed, and where the cure appeared to be complete.

It is an important question, *How do veins once obliterated become again permeable to blood?*—Phlebitis obliterates veins: 1. By infiltration of serum into their surrounding cellular tissue, and into the substance and on the surface of their proper tunics. 2. By the secretion of organizable matter in the same parts. 3. By coagulation of blood. It is evident that a diminution of inflammation will account for the re-establishment of the current of blood. But M. Davat asserts, that by his method an inflammation is excited, which gives rise to adhesion of the opposed venous surfaces, by a fibrous tissue, and that this adhesion is indestructible, as is shown by experiments on dogs. But it is not certain that the adhesion which takes place in the veins of dogs will also occur in varicose veins. These no longer possess their normal texture; they are changed into a fibrous tissue, having but little disposition to secrete organizable matter, and consequently contract to adhesions; the inflammatory phenomena occur rather in the healthy cellular tissue around than in the substance of the veins themselves. These considerations explain clearly the want of adhesion, and the merely temporary obliterations which, at first sight, appear somewhat unaccountable.

*Varices treated with Caustic Potass.*—Caustic has been long employed in the treatment of varicose veins; but the principles of its correct application have not been laid down. The following are the rules which have guided me in the treatment of varix by caustic potass:

1. *It is necessary to apply several morsels of caustic potass on the course of the dilated vein, and at a distance of three or four inches from each other.* This is a repetition of the principle already laid down in speaking of the treatment by means of pins.

2. *The caustic potass should only be applied to the veins at such points as these correspond to the muscles.* Other situations than these are unfavorable to cicatrization, and if cicatrization takes place, the ulcers are readily renewed. The situations whieh I prefer, are,—1, for the thigh, at the height at which cauteries are commonly applied, but a little backward, in consequence of the situation of the vein; 2, for the leg, at the height commonly chosen for cauterization; 3, the middle of the thigh, or the middle of the leg, if three applications should be necessary. In each case, it is supposed that the vena saphena is alone affected.

3. *It is necessary to apply the caustic at least twice, in order to reach the vein.* This supposes, that in order to obliterate the veins, it is necessary to open them by the caustic. All the cases which I have observed, have convinced me that this is a fact. The undulation of blood has never ceased after the first application, which has only implicated the skin and subjacent cellular tissue. The conversion into a hard and impermeable cord has never happened, until after a second application. Would it be better to destroy sufficient at once, or by making two successive applications, to make the second application in the centre of the eschar produced by the first? The reply is not doubtful, if it be considered that it is sufficient to open the vein, and to destroy it to the extent of a few lines, and that any further destruction is useless. A single piece of caustic, which would reach the vein, would make a large eschar of the integument; but with a small piece, twice applied, the part may be hollowed out without extensive destruction of the surface. We should wait three or four days, before applying the second piece of caustic. The plan then is, to make a crucial incision through the eschar, and to insert the caustic into this incision. After the second application, the blood escapes. It is possible, that even a third application may be required to open the vein, but this has always been sufficient. In this way I have applied the caustic, both when I employed it alone, and when I conjoined with it the use of pins. I have employed this treatment in fourteen cases, with different results as it regards the cure of the disease, but always without serious effects.

We pass over M. Bonnet's observations on the cases in which he employed both caustic and pins, because he gives the preference to the treatment by the former alone.

*Caustic potass applied over veins does not expose the individual to phlebitis*, i. e. to that kind of phlebitis which extends from the part operated on towards the trunk. Of course a certain amount of local inflammation is essential to the cure. In advancing the above proposition, I am supported by the fourteen cases in which the potass was employed alone, and by six others where it was combined with pins. Here are twenty cases, in none of which was the inflammation disposed to pass along the veins, although three or four applications of caustic were made in their course, most of which opened their cavities. The patients were simply confined to bed, taking their usual diet at the same time. General facts are also in support of the opinion which I derive from my experience. These facts show that cauterization limits all inflammations which are disposed to extend.

*The application of caustic potass is followed by a circumscribed inflammation, and by ulcerations which are slow to cicatrize.*—In the cases where several pieces of caustic are applied very near each other, the inflammations which surround each eschar run together, and the consequence is a true phlegmon.

*The application of caustic potass exposes to hemorrhage; but this hemorrhage may be easily avoided, if the patient keeps his bed, and if a slight compression is exercised around the limb.*—It has been already said, that it is necessary to apply the caustic until a slight escape of blood shows that the vein has been opened. There may be an actual hemorrhage instead. How long should a patient remain in bed, to render him safe from all chance of hemorrhage? This cannot be fixed by days, but must depend on the condition of parts. When the vein is opened, and this is known by the escape of a few drops of blood, the blood coagulates beneath and above the perforated part; the vein becomes hard, and percussion communicates no undulation to the blood. These signs, which demonstrate the obliteration of the vein, show that there is no further chance of hemorrhage. Four or five days commonly suffice for the accomplishment of these phenomena. In order to second the effects of rest, a bandage should be applied around the limb, immediately after the application of the second piece of potass. The neglect of this was, in one case, followed by hemorrhage, one hour after the application of the second caustic. But a roller immediately stopped the bleeding. No other patients treated with caustic potass, who kept their bed, and to whose limbs a roller was applied, suffered from any hemorrhage worthy of the name; the effusion of a few drops of blood alone announced that the vein was opened.

*In the varices which are limited to the internal saphena and its divisions, and which affect persons within sixty years of age, caustic potass produces a complete and permanent cure.*—The fact of the completeness of the cure rests on fourteen cases. Those which are related, leave no doubt of the superiority of cauterization compared with the employment of pins, as a mean of effecting the obliteration of veins. In one case only was there not a complete interruption to the current of blood in the vein.

*The cure of varices by caustic potass, or by any other means, should not be attempted when the internal and external saphena veins are dilated.*—In the only two cases in which the caustic was applied to varices of both saphenæ, it was observed, that whilst the internal saphena was obliterated, the external saphena acquired an increase in size, and that varices previously but little apparent, became more voluminous, so as to substitute a new disease for that which had been cured. Advanced age, and the thickness of the coats of veins, such as renders their approximation difficult, even with the pressure of the finger, are likewise circumstances unfavorable to the use of caustic potass.

*The treatment of varices by caustic potass hastens the cicatrization of ulcers which co-exist with them.*—This may in part be explained, by the revulsion caused by the numerous artificial ulcers, and by the cure of the varices.

*In what cases should cure of varicose veins be attempted?*

1. Whenever varices ulcerate, and give rise to hemorrhage.
2. When varices exist with ulcers so extensive as to require rest of six weeks, two months, or upwards.

Setting aside these complications, I think we should attempt to diminish the swelling by a stocking. If there are no ulcerations, or if these are but little extensive, if they may get well in one or two weeks, it is unnecessary to keep the patient in bed for above a month; this time is necessary for the separation of the eschars formed by caustic potass, and for the cessation of the pain which remains for some time afterwards in the deep ulcerations. In this case, the remedy would indeed be worse than the disease. M. Bonnet concludes the above highly interesting and useful memoir, by an eulogium on the superiority of the treatment therein recommended over every other, and by a prediction that it will gain the credit which it deserves.

*Archives Générales de Médecine. Mai, Juin,* 1839.

---

*Neuralgia of the Inferior Dental Nerve cured by Section and the Actual Cautery.* By Dr. Sani.

G. L., aged fifty, of a delicate habit, was brought to a state of extreme sensibility, by frequent child-bearing, and various severe diseases. At the cessation of menstruation she was seized with neuralgia of the inferior dental nerve. The pain began at the left mental foramen, with torpor and formication, and very quickly became pulsatile and extremely acute, darting like a flash to all the branches of the nerve, and then extending to all the other divisions of the fifth pair, and of the cervical plexus, with which they are connected. There were redness of the skin, and contractions of the muscles corresponding to the parts that were in pain; the secretion of saliva was increased, and there was difficulty in speaking and masticating. The attacks of pain, which were at first slight and occasional, became intense and very frequent, in spite of the various means that were employed; e. g. anodynes, leeches, extraction of the teeth, tonic and astringent lotions, &c.; and the patient lost her rest and her appetite, and became emaciated.

Dr. Sani, therefore, decided on dividing the nerve. An incision was made along the fold that connects the lip to the gum, from the fourth molar to the first incisor tooth, and was continued in depth till it exposed the inferior dental artery and nerve emerging from the mental foramen. Some attempts were made to separate them, but in vain; they were therefore both divided together, close to the foramen, and a portion of each, two or three lines long, was removed. The division of the nerve caused the most acute pain, and hemorrhage took place from the artery, to which it was found necessary to apply the actual cautery. The simplest means were afterwards employed. On the eighteenth day, the cicatrization was complete, and, except for some slight creeping pain, which the patient felt from the eighth to the eleventh day after the operation, no inconvenience was afterwards suffered. The parts supplied by the divided nerve remained completely insensible, but their motions were unimpaired.

*Bulletino delle Scienze Mediche. Gennajo,* 1839.

---

*Case of Idiopathic Mydriasis.* By Dr. Kochanowski.

The dilatation of the pupil was in this instance so considerable, that the iris formed only a very narrow ring. The strongest light produced no effect on it. After unsuccessfully trying a variety of medicines, it struck Dr. Kochanowski that the ergot of rye might be useful. On the twenty-second day of the disease, if it may be so termed, the exhibition of this medicine was commenced, in doses of three grains four times daily. On the following day, the patient (a woman aged thirty-three, long subject to hemorrhoids and menstrual derangement,) became conscious of a change in the state of the part; she felt something move, as it were, in the eye, under the influence of light. On examination, the pupil was found to have contracted a little. The dose was now increased to fifteen grains: the improvement advanced. During the menstrual flow the administration of the drug was discontinued, and the dilatation of the pupil returned. After the disappearance of the menses, the dose was raised to eighteen grains, or a scruple; at the end of a few days the pupil had recovered its normal dimensions, and the iris its usual contractility.—(*Memoirs of the Medical Society of Warsaw.*) *L'Expérience. Septembre,* 1839.

---

*On a new Method of Making an artificial Anus in the Lumbar Region.*
By M. AMUSSAT.

A QUESTION which has lately been exciting considerable interest among the surgeons in Paris is, the practicability of forming an artificial anus in the lumbar region, in cases where a cancerous or other disease causes an insurmountable obstruction to the natural course of the fæces. Callisen recommended this operation, but its propriety has been generally disputed by modern surgeons. M. Amussat, however, meeting with a patient in whom such an operation was required, has revived Callisen's proceeding, with some modifications, and has successfully practised it in two cases.

The first case was that of a woman, forty-eight years of age, who had been long subject to constipation. In the beginning of May, 1839, the constipation became more obstinate than usual, and resisted the use of baths, injections, and the most active purgatives. After the ineffectual employment of these remedies for twenty-six days, a consultation was determined on, and MM. Barras, Amussat, Fouquier, Breschet, Récamier, and Puyoo, were called in. On examination of the rectum, a hard, round, and almost immovable tumour was felt at a considerable height in the intestine, about double the size of the neck of the uterus. The only resource, in this case, seemed to be the formation of an artificial anus, which was accordingly made on the 2d of June, by M. Amussat, in the following manner: The patient was placed on her face, with her abdomen supported by pillows; and a transverse incision was made in the left lumbar region, where the accumulation of fecal matters caused a considerable projection. The incision was commenced at the external edge of the sacro-lumbalis and longissimus-dorsi muscles, and was extended outwards for four inches and a half, at the distance of two fingers' breadth above the crista of the ilium. The skin and muscles being divided, the adipose tissue surrounding the kidney was brought into view, on which the posterior part of the lumbar colon rests: on cutting through this fat, the colon immediately appeared between the edges of the wound, in a very distended state. A loop of thread having been passed through the most projecting part, for the purpose of holding it in its situation, a trocar was first plunged into the intestine, when a quantity of gas and liquid fæces escaped through the canula. An incision of an inch and a half in extent was then made with a bistoury, in the transverse directionof the colon, and an immense quantity of fæces was discharged, the exit of which was encouraged by injections of warm water into both ends of the intestine. The edges of the wound in the colon were, lastly, fastened by stitches to the margins of the most anterior part of the external wound, and the whole was covered with a poultice.

Very little hemorrhage occurred during the operation, and the relief of all the symptoms was immediate, the patient passing a good night afterwards. On the following day, slight symptoms of inflammation showed themselves, but they were removed by the application of some leeches. The external wound rapidly healed, and, at the period of four months after the operation, the patient was quite well, and had regained her strength; the artificial anus presented a regular rounded orifice, through which the fecal matters passed in a solid form, once or twice a day. A simple bandage fixed round the body was sufficient to retain them, which was taken off when an inclination to void the fæces was felt. Nothing, except gas, passed by the natural anus. [No mention is made of the state of the disease of the rectum, after the operation.]

The other case was very similar to the preceding. The operation was here formed on a man, aged sixty-two, who was affected with cancer of the rectum, which entirely obstructed the canal. This case turned out equally successful; the man's health was much improved by the operation, and the progress of the disease of the rectum seemed to be arrested.

The formation of an artificial anus in the groin, in certain cases, was proposed formerly, by Littre; and eleven cases are on record in which it was performed, of which number six died. Eight of these cases were in infants, and only three in adults, out of whom one died. M. Amussat considers that his operation (which differs from that of Callisen, in the incision being transverse instead of vertical,) possesses several advantages over that of Littre, the principal of which is, that the peritoneum is not wounded; this part of the intestine possessing no mesocolon, according to M. Amussat, in any instance where it is distended with gas or fecal matters. Another advantage of it is, that an artificial anus in the lumbar region is less affected by the contractions of the abdominal muscles than in the groin, and consequently prolapsus of the intestine is not so liable to occur.

[The expediency of performing this operation, in any case, has been questioned, for it has been said that the disease which causes the obstruction in the rectum is almost always of a malignant character, and will, sooner or later, destroy the patient; and the operation, if successful, can only prolong a life of misery for a very short period. These arguments, however, ought to have no influence over the measures of the surgeon, whose duty it undoubtedly is, to prolong life to the greatest extent, under almost all circumstances, particularly in adults, the continuance of whose existence, even for a few months, may be of the utmost value to themselves and families. The propriety of forming an artificial anus in the loins or groin of an infant, in whom the rectum is deficient or malformed, may perhaps be reasonably doubted.]

*Bulletin Gén. de Thérapeutique,* 15 *et* 30 *Oct.* 1839.

---

*Treatment of External Cancer by Ligature of the Vessels and Division of the Nerves supplying the diseased part.* By M. Jobert.

Feeling persuaded that the increased afflux of blood and heightened nervous sensibility, which are the consequences of disease, exert a great local influence in cancerous affections, M. Jobert has adopted a new plan of treatment; namely, that of tying the principal arterial branches and dividing the nervous filaments which are distributed to the affected part. He has seen this proceeding followed by a favorable change in the aspect of the ulcers, and by their ultimate cure. He has obtained this successful result in four cases of cancer of the lip, and in one of the tongue.

M. Jobert is of opinion that the vascular system has a much more important share in the development of cancerous affections than the nerves of the part; therefore he considers that tying the arteries will have much more influence in checking the progress of the disease than the division of the nervous filaments.

*Revue Médicale, Sept.* 1839.

---

*On a new Method of Detecting Changes of Form in the Prostate Gland.* By A. Mercier, m.d.

[M. Mercier having pointed out some defects in the modified catheter employed by M. Leroy d'Etiolles for the above purpose, describes the instrument invented by himself.]

The instrument I have employed for upwards of three years, with marked success, is extremely simple, being nothing more than a sound, which is perfectly straight, except at the vesical end; here it is curved at a right angle at about six or eight lines from the point. Once this sound is introduced into the bladder, it may, on account of the shortness of its curved extremity, be moved about the neck without the least difficulty. The external extremity is provided with an oval or polygonal plate perpendicular to the curved part of the instrument; an index on one of the surfaces of this plate shows on which side the point of the sound lies at any given moment while the operator rotates it. There is no particular difficulty in introducing this sound, especially in old people, who are peculiarly subject to the diseases it is designed to detect. The prostatic portion of the urethra would be the part most likely to present the obstacle to its passage; now Hunter has remarked that in cases of hypertrophy of the lateral lobes, the antero-posterior or recto-pubic diameter of the urethra invariably undergoes considerable increase: I have myself seen it measure fourteen or fifteen lines. When the instrument reaches the prostatic division of the canal, the free extremity must not only be lowered, but pushed straight on towards the neck of the bladder: by these means the point will be prevented from catching against the anterior wall of the canal.

*Method of detecting prostatic tumours protruding into the bladder.*—When the sound has been passed into the bladder, the next thing to be done is to place it in such position that its straight portion shall be nearly parallel to the axis of the body. The curved point is then drawn against the anterior border of the neck of the bladder, and then turned to the right and left, and carried round the circumference of the opening of the urethra. When this surface of the prostate is in a normal state, the point of the sound may be swept round the whole extent without obstruction. But if there be a tumour connected with the neck, the instrument is checked in its progress, and in order to carry it over the obstructing body, it becomes necessary to raise the point proportionally to the size of that body. The plate already referred to shows *on what side* of the prostate the stoppage has occurred: the arc of a circle described in the rise and fall of the point, as it passes over the obstacle, represents the *width* of the tumour, and its *height* is measured by observing at the extremity of the glans how many lines the instrument has risen. Finally, from the greater or less degree of abruptness with which the point rises and falls, we learn whether the prominence is *pedunculated or provided with a broad base.*

[The reader will readily conceive the modifications required in the mode of manipulating, when the neck of the bladder or the prostatic portion of the urethra are to be examined. M. Mercier, after stating these, continues:]

Such are the results obtainable with this instrument. By it we are enabled to discover the organic cause of numerous cases of paralysis of the bladder, erroneously considered essential. The consequence of a correct diagnosis is here of no mean importance, in a therapeutical point of view; for even if the affection we thus discover be regarded as incurable (which is a most serious error,) we shall at least learn the necessity of abstaining from the use of irritating injections, blisters, friction with the tincture of cantharides, &c. For it is evident that a plan of treatment of this kind will be the more seriously injurious the more fully it realizes the effect intended in its employment—the more it increases the contractile power of the bladder, which can only exhaust itself in vain efforts to overcome a permanent obstacle.

The instrument is also available in the diagnosis of tuberculous disease of the prostate occurring in youth, of inflammatory swellings and abscesses, and in the discovery of calculi concealed behind prominences of the neck of the bladder.

*Archives Génerales de Médecine. Juin,* 1839.

